# The New Editors of the Radium Department

**Published:** 1916-09

**Authors:** 


### EDITORIAL DEPARTMENT

MRS. F. E. DANIEL, Publisher and Managing Editor.

### The New Editors of the Radium Department.

Since the discovery of radium by the Curries in 1889, and the discovery of X-rays by Professor W. C. Roentgen in 1895, the interest in these subjects has grown steadily, until today quite a number of physicians are devoting their entire time to this work; indeed, so broad is the field that specializing becomes a necessity, if one would do good work.

Believing it to be the duty of all physicians to keep informed upon all subjects, and knowing that the JOURNAL'S readers desire to do so, we are this month adding a department of X-ray and Radium Therapy, which shall be edited in such a practical and helpful way as to enable the general practitioner to diagnose, readily, cases in need of such treatment.

Dr. Edward H. Skinner, of Kansas City, Missouri, will have supervision of this department.

Dr. Skinner has been doing X-ray work since 1904, and has specialized in Roentgenology since 1909. He spent the winter of 1909 and 1910 in Europe doing special work in radium with Wickman, of Hamburg, and Albers Chamburg at St. George Krankenhaus.

One of the most prominent and best loved physicians in Texas in writing to congratulate the Journal upon having secured the services of Dr. Skinner said: "I am indeed glad that you have succeeded in securing the services of Dr. Skinner as editor of the Radium Department of your JOURNAL. I know Dr. Skinner well, and I can assure you that aside from being a truly scientific man he is also a Prince among men, and because of his wonderful strides in the scientific world, due solely to his energy and ambition, he is worthy of the highest positions of honor and trust that can be bestowed upon him."

We take pleasure and pride in introducing to you Dr. Edward H. Skinner.

Dr. J. M. Martin, of Dallas, Texas, needs no introduction, as a physician, to the medical profession of Texas. As assistant editor of the Radium Department of the TEXAS MEDICAL JOURNAL he is presented to you at this time.

Dr. Martin was born in Missouri, studied medicine in Valparaiso, Indiana, and St. Louis Medical College, from which college he received his degree in 1892.

Very early in his career he became interested in electrotherapeutics. In 1905 Dr. Martin was elected to the chair of Electrology and Radiology in the Medical Department of Baylor University. This position he still holds.

With a view to aiding beginners in this most difficult work, the doctor compiled and published in 1912 a book on "Practical Electrotherapeutics and X-Ray Therapy." The book has proven very popular with those interested in this subject.

At present the doctor's entire time is devoted to diagnostic and therapeutic uses of electrology, radiology and radium therapy, and everyone who knows his height—six feet four inches—accords him the honor of being the "highest" authority, in Texas, upon the subject of X-ray and radium therapy.

